# YOLO-CBNet: A robust attention-enhanced detection framework for underwater fish recognition in aquaculture environments

**DOI:** 10.1371/journal.pone.0341525

**Published:** 2026-09-15

**Authors:** Mahdi Hamzaoui, Leila Bousbia, Mohamed Ould-Elhassen Aoueileyine, Imen Filali, Ridha Bouallegue

**Affiliations:** 1 Innov’COM Laboratory, Higher School of Communication of Tunis, University of Carthage, Technopark Elghazala, Raoued, Ariana, Tunisia; 2 Department of Computer Sciences, College of Computer and Information Sciences, Princess Nourah bint Abdulrahman University, Riyadh, Saudi Arabia; Mansoura University, EGYPT

## Abstract

The accurate identification of fish species remains a pivotal challenge in aquaculture, as it directly influences population management, health monitoring, and overall production efficiency. Yet, underwater environments often present difficult conditions such as reduced visibility, suspended particles, and uneven lighting that significantly limit the performance of traditional recognition approaches. In this study, we introduce YOLO-CBNet, an enhanced architecture designed to overcome these constraints and provide more dependable monitoring in real-world aquaculture systems. The method adopts a two-stage strategy. First, a Contrast Limited Adaptive Histogram Equalization (CLAHE) module is employed to counter low contrast, turbidity, and color distortion, restoring essential visual information. Second, the YOLOv11 framework is refined through the integration of the Convolutional Block Attention Module (CBAM) and a Bidirectional Feature Pyramid Network (BiFPN), a combination intended to improve multiscale feature fusion and reinforce the detection of small or partially occluded fish. Experimental results indicate that YOLO-CBNet delivers a clear performance boost, the precision score reaches 0.951, surpassing the baseline YOLOv11 model, which achieved 0.911 under the same conditions. These improvements highlight YOLO-CBNet’s potential as a reliable solution for underwater detection and recognition tasks within modern aquaculture environments.

## Introduction

In the past, fishing solved major problems related to the food crisis. But increasingly, production in this sector is declining due to climate change. Aquaculture, a method of farming aquatic species, is a reliable solution to prevent shortages of aquaculture products. According to the FAO, since 1961, global consumption of products (excluding algae) has been growing at an average annual rate of 3.0%, twice as fast as the annual growth of the world’s population. Consumption now stands at 20.2 kg per person, more than double the level observed in the 1960s. In 2020, aquatic animal production was 30% higher than in 2000 and over 60% higher than the average recorded during the 1990s. Despite the onset of the COVID-19 pandemic, 157 million tons (89%) of aquatic animal production in 2020 was destined for direct human consumption [1].

In the aquaculture sector, underwater monitoring is particularly challenging due to the inherent characteristics of aquatic environments. Water turbidity and low illumination significantly degrade image quality, which complicates fish detection and classification tasks [2,3]. Moreover, the visual similarity between fish skin textures and the surrounding background further hinders accurate identification [4]. These difficulties are amplified by the continuous movement of fish schools, which often leads to overlapping and occlusions, reducing the effectiveness of conventional detection methods [5]. Consequently, robust automated monitoring systems based on efficient and adaptive computer vision and machine learning techniques are essential to ensure reliable fish behavior analysis and optimal aquaculture management.

Traditional automated fish monitoring methods still face significant limitations in aquaculture environments, mainly due to the unstable and complex conditions inherent to underwater scenes. The processing of underwater images and videos is particularly challenging because of turbidity, variable illumination, and surface reflections. In such dynamic conditions, conventional approaches often struggle to maintain robustness, especially under rapidly changing lighting and the continuous movement of fish schools. These limitations reduce their reliability for accurate and real-time monitoring. In contrast, recent advances in machine learning provide more effective and adaptable solutions, offering improved performance in complex underwater environments. Incorporating deep learning techniques with computer vision now makes it possible to develop accurate tools for fish detection, weight prediction, disease identification, and animal welfare assessment. These hybrid and adaptive methods outperform traditional methods in terms of accuracy and robustness, thus providing the ability to process massive amounts of video data in near real-time, which is considered a major asset for efficient aquaculture management. This paper proposes a study that explores the performance of YOLO enhanced with the Convolutional Block Attention Module (CBAM), a lightweight attention mechanism that sequentially infers channel and spatial attention to emphasize informative features, and the Bidirectional Feature Pyramid Network (BiFPN), a feature fusion strategy that efficiently combines multi-scale features in both top-down and bottom-up pathways, aiming to achieve accurate detection of fish in underwater environments. A review of previous work and an analysis of related results are presented in Section 2. Section 3 provides details on the dataset understanding phase and the preprocessing techniques applied. We also describe the training process of the proposed YOLO-CBNet architecture, emphasizing the integration of CBAM and BiFPN to improve feature representation and multi-scale detection. Section 4 presents the experimental results and evaluates the performance of the YOLO-CBNet model in comparison with existing approaches. Finally, Section 5 concludes the study and outlines potential directions for future improvements.

### Related work

Fish detection and classification are fundamental tasks leading to productive aquaculture. Several previous works have focused on improving models related to this field of activity.

Early approaches to fish detection in underwater environments rely on classical computer vision methods such as segmentation based primarily on color or contours, adaptive thresholding, and background difference [6–8]. These techniques are simple to implement with low computational costs, but they lack robustness in the face of underwater complexity [9]. In addition, they often require precise calibration for each aquaculture site, which limits their generalization.

Improving the quality of underwater images is a major challenge. Much work has focused on this field to improve the models robustness. With the advent of CNNs (convolutional neural networks), several studies have shown significant advances in fish detection and classification. For example, Wang et al. [10] presented a high-accuracy real-time fish detection model based on self-build dataset and RIRD-YOLOv3, with a clean dataset, using an image recovery phase to mitigate the effect of noise and poor lighting before applying You Only Look Once version 3 (YOLOv3). The results obtained are around 0.85 for the mAP metric. However, the proposed method was evaluated on a relatively controlled aquaculture dataset, which may limit its generalization to more challenging underwater environments. In addition, the architecture does not incorporate advanced attention mechanisms or multi-scale feature fusion strategies, which are important for detecting small and partially occluded fish. In another study, Chen et al. [11] proposed a lightweight method for spatially counting intensive fish schools in fish farming environments, based on an improved version of YOLOv5. This highlights the importance of efficient real-time methods adapted to complex and varied conditions. Despite these promising results, its evaluation focuses mainly on fish school monitoring in aquaculture environments, which may limit its generalization to broader underwater scenarios involving multiple species, complex backgrounds, and severe visual degradations. Rabhavathy et al. [12] opted for a combination of attention modules (CBAM), Swin transformers, and diffusion models to improve the quality of underwater images, achieving a detection accuracy of 81.4% on the TrashCan dataset. Although the proposed framework significantly improves underwater image quality and detection performance, its evaluation is limited to the TrashCan dataset and focuses primarily on underwater object detection rather than fine-grained fish species recognition in diverse underwater environments. Liu Zhuoyan et al. [13] developed a plug-and-play module for detector models such as YOLOv5, trained without additional data, which improved detection by up to +3.3 Average Precision on the URPC2021 dataset. Although this approach shows good generalization and plug-and-play capability, its evaluation is mainly limited to benchmark datasets, and its effectiveness for fine-grained multi-species fish detection in complex underwater environments is less explored. Guan et al. [14] proposed AUIE-GAN, an adaptive underwater image enhancement model based on generative adversarial networks. This adaptive GAN model is trained on UIEB and SUID datasets, outperforming other methods such as FUnIE-GAN (Fast Underwater Image Enhancement GAN) and PUIE-Net (Perceptual Underwater Image Enhancement Network) in terms of objective measures and visual quality. While showing strong performance in underwater image enhancement, this GAN-based method remains computationally expensive and is mainly validated on limited datasets, which may restrict its real-time applicability in underwater detection systems. To address the challenges posed by environmental variability in underwater imaging, Huang et al. [15] combined a decision tree with a support vector machine (SVM). The model was trained on 24,000 images distributed across 15 fish categories and achieved an accuracy of 74.8%. They proposed a novel deep neural network to further improve performance, due to its superior ability to capture complex patterns and adapt to data variability.

CNN-based methods have achieved encouraging results in underwater environments due to their ability to extract complex image features and handle variability in shapes, textures, and environmental conditions. Valle et al. [16] presented a new deep learning model for fish recognition and length estimation, addressing challenges related to texture and shape similarity between fish species. To improve image segmentation, they tuned the Mask R-CNN algorithm and used MobileNet-V for length estimation. Although multiple datasets were used, COCO served as the basis for the pre-trained model, which was not optimized for specific identification tasks. Deep et al. [17] used the Fish4Knowledge dataset to improve image quality using sharpening techniques. After analyzing various algorithms, they demonstrated that the hybrid DeepCNN-KNN approach provided superior performance, with an accuracy of up to 98.79%. The method achieves high accuracy on the Fish4Knowledge dataset using a hybrid DeepCNN-KNN approach; however, its reliance on controlled dataset conditions and limited evaluation of real-world underwater variability may restrict its generalization capability.

The single-stage detector architecture, known as YOLO, has attracted considerable interest due to its strong performance in marine species detection. EnYOLO merges image enhancement and object detection within a unified framework, facilating deployment on low-resource autonomous underwater robots [18]. Zhang et al. [19] present a new lightweight method for underwater object detection combining MobileNet v2 and YOLOv4. The architecture integrates depth-separated convolutions and an improved Attentional Feature Fusion (AFFM) module. This approach significantly minimizes the model size and parameter count while maintaining good accuracy. Experimental work shows an effective trade-off between speed (44.22 FPS) and accuracy (mAP of 92.65% on the Brackish dataset), making the proposed solution suitable for real-time applications. Zhao et al. [20] propose an improved solution of YOLOv4-tiny called YOLO-UOD, for real-time underwater object detection with low computational cost. They insert a dilated symmetric bottleneck, an FPN-Attention module combining spatial and channel-based attention, and a label smoothing strategy to enhance feature fusion and accuracy. On the brackish dataset, YOLO-UOD achieves an mAP of 87.88% and runs at 9.24 FPS on Jetson Nano 2GB, outperforming YOLOv4-tiny and YOLOv5. Lawal et al. [21] presented a lightweight underwater marine species detector based on the YOLO framework and combining anchor-based and anchor-free techniques. The detector is named MADNet and it integrates the modules CBS, C3b, Bottleneck, SPPFr, and C3. MADNet outperforms several lightweight YOLO versions such as YOLOv5n, YOLOv6n, YOLOv7-tiny, and YOLOv8n in accuracy, speed, and computational cost, with a performance score of 27.8%. This method offers an effective solution for fish detection in harsh underwater environments and on low-power onboard devices, but its evaluation is primarily focused on benchmark comparisons and may not fully capture performance in highly complex real-world underwater scenarios with severe occlusion, turbidity, and illumination variations. Tang et al. [22] proposed a shrimp-like underwater bionic robot that contains an improved MGC-YOLO model based on YOLOv8s, incorporating Multi-scale Ghost convolutions and the CPMS attention module for foreign body identification. The robot combines mobility, grasping, and buoyancy, and can be controlled via an app with real-time image transmission. The experiments performed showed a significant reduction in parameters and computation, as well as an improvement in accuracy and mAP, confirming the effectiveness of the system for underwater foreign body recognition.

Despite the strong performance of CNN-based methods, they still present limitations related to their limited capacity to capture long-range dependencies and model complex variations in shape and texture [23]. Vision transformers have emerged as an effective solution to these limitations by leveraging attention mechanisms to capture global contextual relationships and improve generalization across varying conditions [24]. Ji et al. [25] presented IMViT, an innovative method for fish detection and classification. They used the traditional ViT model and integrated convolutional and residual reinitialization units to improve detection and classification capabilities. The proposed model achieved promising results, even with small datasets. Peng et al. [26] created the LSUI dataset, a large set of high-quality underwater images, segmentation maps, and transmission maps, to address the problems related to the lack of diverse data. They present a U-shaped Transformer network combining the CMSFFT and SGFMT modules, suitable for underwater image enhancement by taking into account spatial and channel-specific attenuations. Experimental work has shown that the proposed method outperforms existing performance by more than 2 dB, thus improving contrast and saturation according to the principles of human vision. However, the method is primarily evaluated on image enhancement benchmarks and does not explicitly address downstream underwater object detection tasks or real-time deployment constraints in practical marine applications.

## Materials and methods

In this section, we present the dataset, the data distribution, and some processing steps involved, including cleaning and bounding box generation operations. We then explain in detail the architecture of our YOLO-CBNet approach.

### Dataset

The dataset used in our study is reconstructed from another public dataset. The images were captured at the fishing port of Cabuyao City, Laguna, Philippines. This newly reconstructed dataset is named “YOLO-ViT dataset.” It contains 1,805 fish images acquired under a variety of environmental conditions. As shown in the Table 1, The dataset is well balanced across the 10 fish species, with a similar number of samples per class, ensuring that the model is not biased toward any dominant category.

**Table 1:** Distribution of fish species in the dataset.

Species	Scientific Name	Number of Images
Bangus	*Chanos chanos*	181
Big Head Carp	*Aristichthys nobilis*	180
Black Spotted Barb	*Puntius binotatus*	179
Climbing Perch	*Anabas testudineus*	182
Fourfinger Threadfin	*Eleutheronema tetradactylum*	180
Glass Perch	*Ambassis vachellii*	181
Gourami	*Trichopodus trichopterus*	179
Jaguar Gapote	*Parachromis managuensis*	181
Scat Fish	*Scatophagus argus*	181
Tilapia	*Oreochromis niloticus*	181

**Fig 1:**
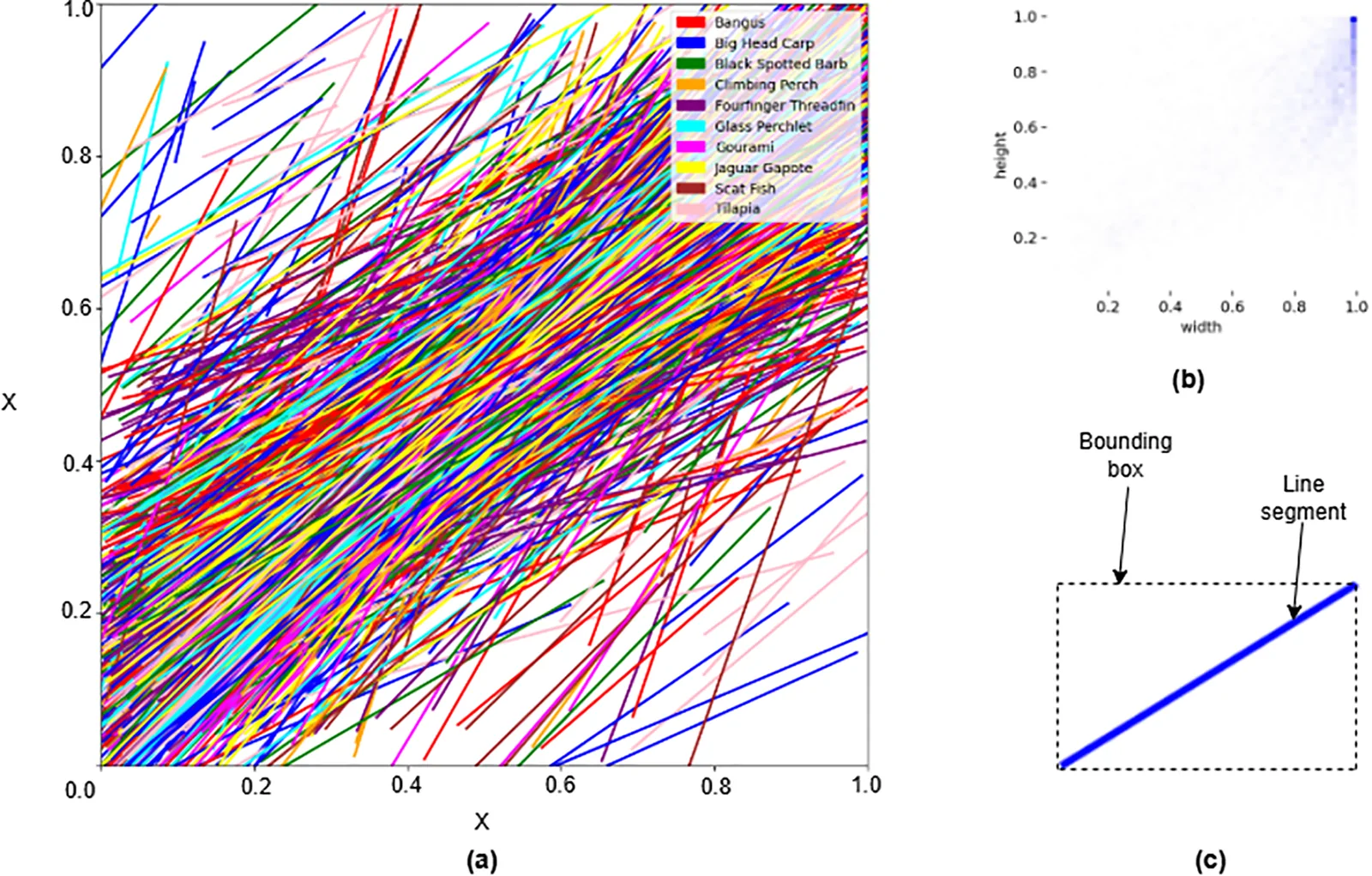
(a) Distribution of normalized bounding-box coordinates across the training set. (b) Distribution of bounding-box widths and heights, highlighting the variability of object scales. (c) Illustration of a bounding box enclosing an object segment.

**Fig 2:**
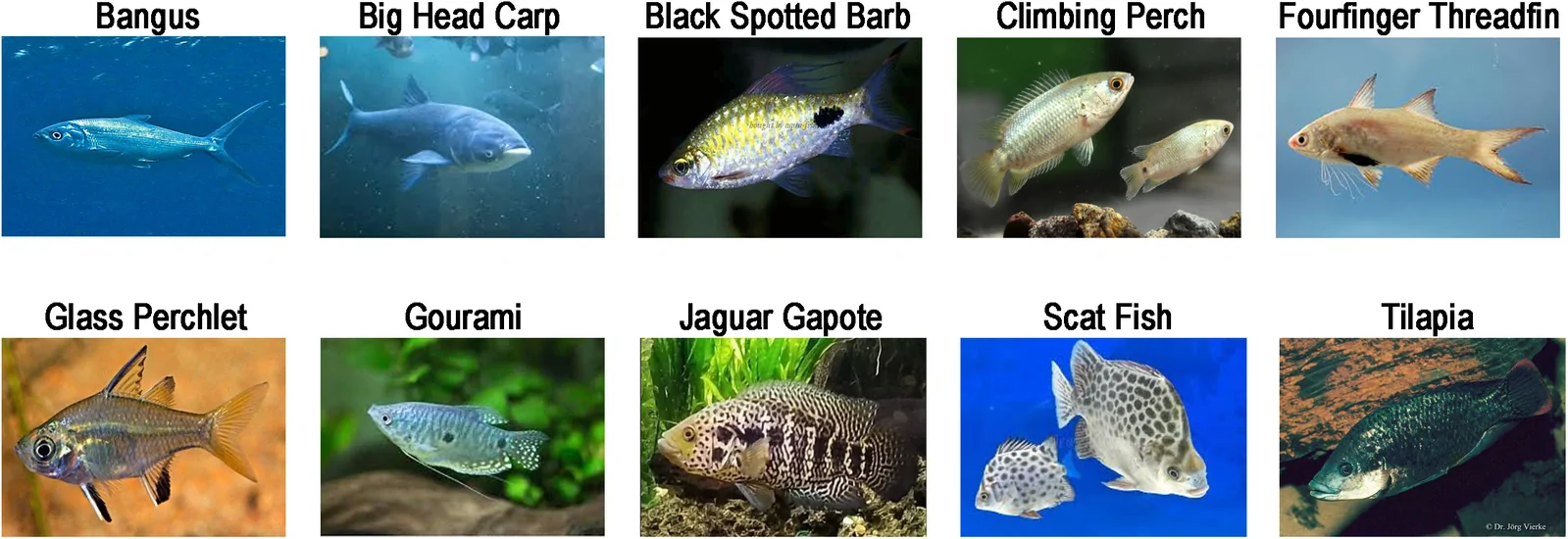
Representative sample images of fish species from the YOLO-ViT dataset, illustrating the diversity in appearance, scale, illumination, and background conditions.

Preprocessing and data augmentation techniques were used. Rotation simulates variations in real-world viewing angles and allows the model to better adapt to real-world image viewing conditions. A rotation between −15° and +15° was applied. Images captured from underwater environments often suffer from attenuation and brightness, which minimizes contrast. To mitigate this issue, grayscale conversion was applied as a data augmentation technique during training, rather than as a preprocessing step on the entire dataset. This transformation helps the model become less sensitive to color variations and improves its robustness under challenging visual conditions. Dataset annotation and the addition of bounding boxes were performed manually using the Roboflow platform. Fig 1(a) presents the coordinates relative to the bounding box positions across all images found in the training set. Fig 1(b) illustrates the normalized distribution of bounding-box widths and heights across the dataset. This visualization highlights the most frequent object sizes and provides insight into the coherence and variability of the annotations. Fig 1(c) shows a segment and its bounding box. Fig 2 presents sample images of fish selected from the dataset representing different species.

### Proposed approach

**Fig 3:**
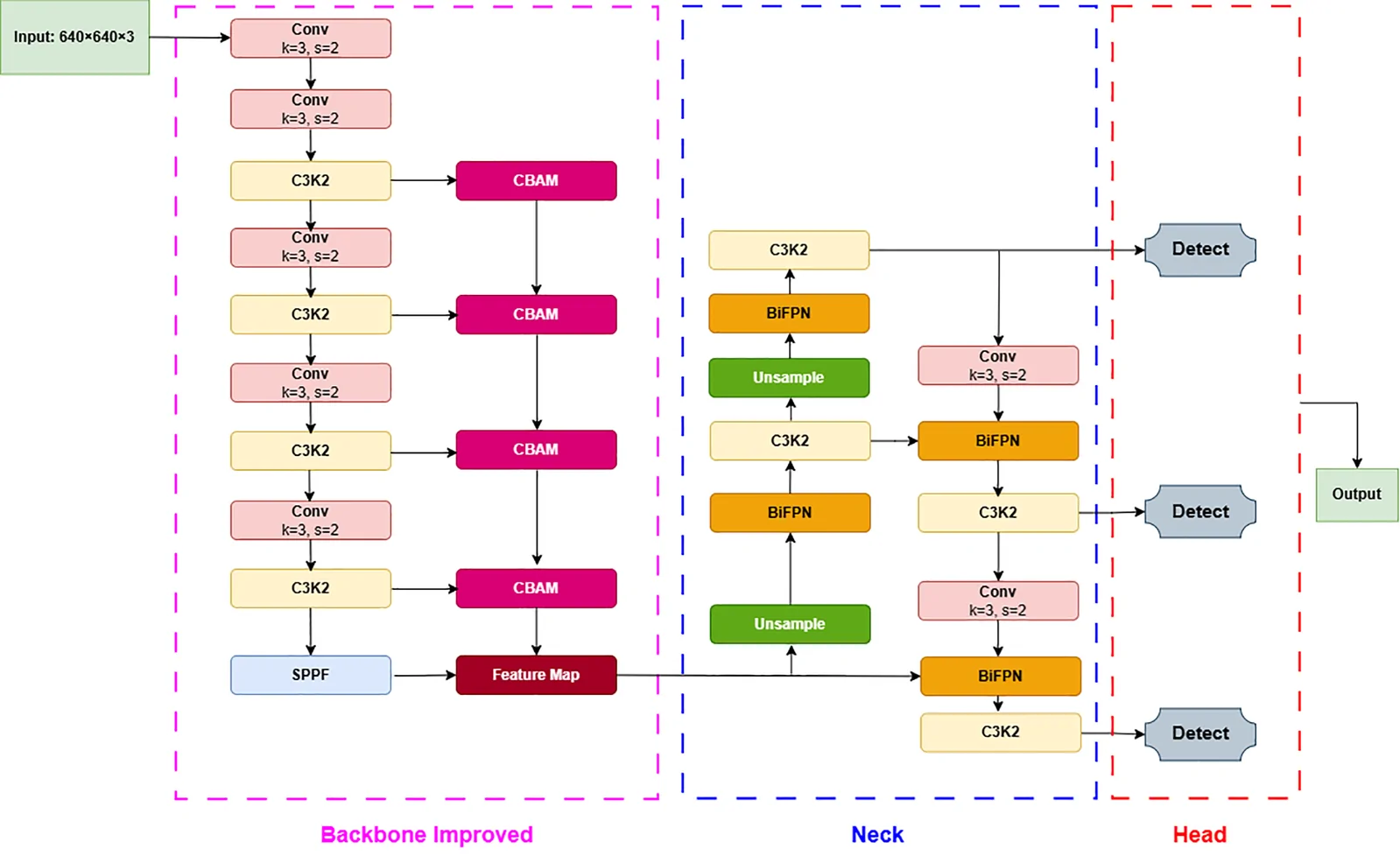
Architecture of the proposed YOLO-CBNet model, highlighting the improved backbone with CBAM attention modules, the BiFPN-based neck for enhanced multi-scale feature fusion, and the detection head for accurate object localization and classification.

As illustrated in Fig 3, the proposed method introduces a task-driven integrated enhancement framework specifically designed for underwater fish detection under challenging visual conditions. The approach consists of two complementary stages. In the first stage, a CLAHE-based preprocessing module is applied to enhance image quality by improving local contrast and mitigating the effects of turbidity, low illumination, and color distortion, thereby facilitating more reliable feature extraction. In the second stage, the YOLOv11 architecture is systematically enhanced through targeted structural modifications. More specifically, the backbone is improved by integrating CBAM attention modules to refine intermediate feature representations by emphasizing informative channels and spatial regions. In addition, a BiFPN-based neck is introduced to enable adaptive multi-scale feature fusion, improving the detection of small, overlapping, and partially occluded fish. Overall, these components are strategically integrated at different levels of the architecture to progressively enhance feature quality and robustness for underwater object detection.

### Backbone enhancement details

**Fig 4:**
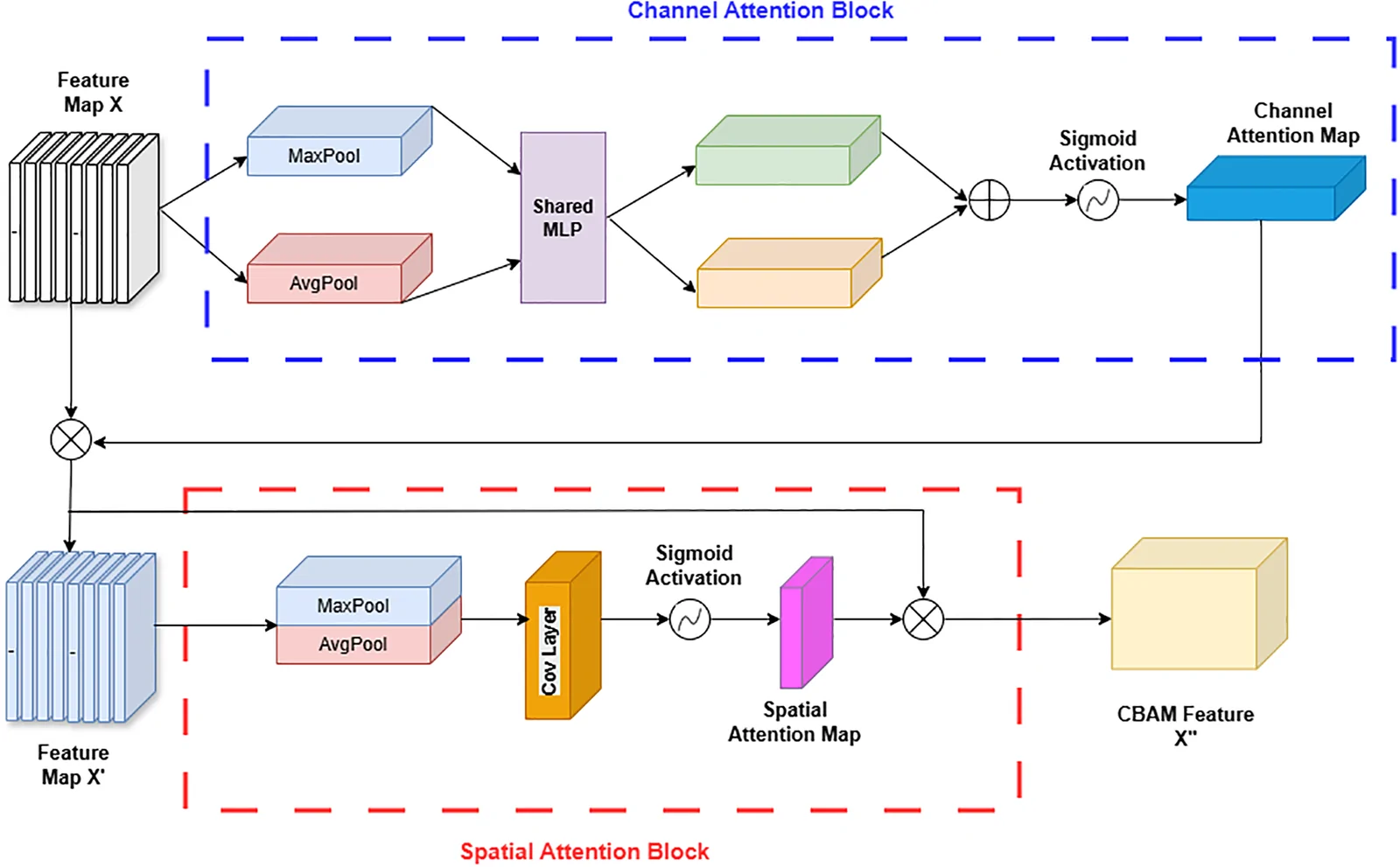
Architecture of the CBAM module, highlighting the sequential Channel and Spatial attention blocks that refine input features by prioritizing relevant channels and localizing salient spatial regions for improved detection accuracy.

As illustrated in Fig 4, CBAM is a lightweight, modular attention module that adjusts feature maps by successively exploiting two types of dependencies: inter-channel and spatial [27]. CBAM consists of two consecutive blocks applied to an input feature map $F\in {\mathbb{R}}^{C\times H\times W}$.

The Channel Attention (CA) Block emphasizes informative channels by aggregating spatial information using both average and maximum pooling operations. Formally, the channel attention map $M_{c}\in {\mathbb{R}}^{C\times 1\times 1}$ is computed as:


$$\begin{array}{c} M_{c}(F)=\sigma (\;\text{MLP}(\text{AvgPool}(F))\;+\;\text{MLP}(\text{MaxPool}(F))\;) \end{array}$$ (1)

where MLP(·) denotes a shared multi-layer perceptron, and σ(·) is the sigmoid function. The refined feature map after channel attention is obtained by element-wise multiplication:


$$\begin{array}{c} F^{\prime }=M_{c}(F)\;⊙\;F \end{array}$$ (2)

The Spatial Attention (SA) Block focuses on localizing informative regions. Spatial attention uses both channel-wise average and max pooling to aggregate channel information into a 2D map, followed by a convolution operation and sigmoid activation. The spatial attention map $M_{s}\in {\mathbb{R}}^{1\times H\times W}$ is defined as:


$$\begin{array}{c} M_{s}(F^{\prime })=\sigma (\;f^{7\times 7}([\;{\text{AvgPool}}_{c}(F^{\prime })\;;\;{\text{MaxPool}}_{c}(F^{\prime })\;])\;) \end{array}$$ (3)

where $f^{7\times 7}$ represents a convolution with a 7×7 kernel, and [·;·] denotes channel-wise concatenation. The final CBAM-refined feature is obtained as:


$$\begin{array}{c} F^{\prime \prime }=M_{s}(F^{\prime })\;⊙\;F^{\prime } \end{array}$$ (4)

Through this sequential attention mechanism, CBAM adaptively highlights the most discriminative channels and spatial regions, effectively reinforcing the feature representation for downstream tasks.

In order to improve the backbone’s feature extraction capacity while maintaining reasonable computational complexity,CBAM modules are strategically inserted after C3K2 residual blocks within the backbone to enhance feature representation while maintaining computational efficiency. This design avoids placing attention after every convolution layer, which would introduce unnecessary computational overhead. Since C3K2 blocks already extract rich local and contextual features, applying CBAM at this stage enables effective refinement through channel and spatial attention mechanisms. This selective integration improves the discriminative capacity of feature maps before propagation to deeper layers. As a result, the final feature maps generated by the improved backbone become richer in information and more relevant for fish detection and classification tasks in underwater environments. This improvement aims to overcome the limitations associated with the complex conditions of the underwater environment, including turbidity, lighting variations, and surface reflections. By enhancing the quality and selectivity of the extracted representations, our approach allows the model to better adapt to these complex and dynamic conditions.

### Neck enhancement details

In order to optimize the fusion of multi-scale features generated by the backbone, we integrate the Bi-directional Feature Pyramid Network (BiFPN) at the Neck level of the architecture [28]. BiFPN is designed to efficiently combine information across different resolution scales, which is critical for accurately detecting objects of varying sizes in complex environments.

Let $\left\{P_{3},P_{4},P_{5}\right\}$ denote the set of feature maps extracted from the backbone at different levels, where $P_{i}\in {\mathbb{R}}^{C_{i}\times H_{i}\times W_{i}}$. BiFPN performs both top-down and bottom-up feature fusion with learnable weights to balance the contribution of each input feature. The fused feature at level *i* can be mathematically expressed as:


$$\begin{array}{c} {\hat{P}}_{i}=\frac{\sum_{j\in 𝒩(i)}w_{j}\;P_{j}}{\sum_{j\in 𝒩(i)}w_{j}+\epsilon } \end{array}$$ (5)

where 𝒩(i) denotes the set of neighboring feature maps for level *i* (including top-down and bottom-up connections), $w_{j}$ are learnable non-negative fusion weights, and ϵ is a small constant to avoid division by zero. The fused features are then passed through a 3×3 convolution layer to generate ${\hat{P}}_{i}$ with refined spatial and semantic information.

**Fig 5:**
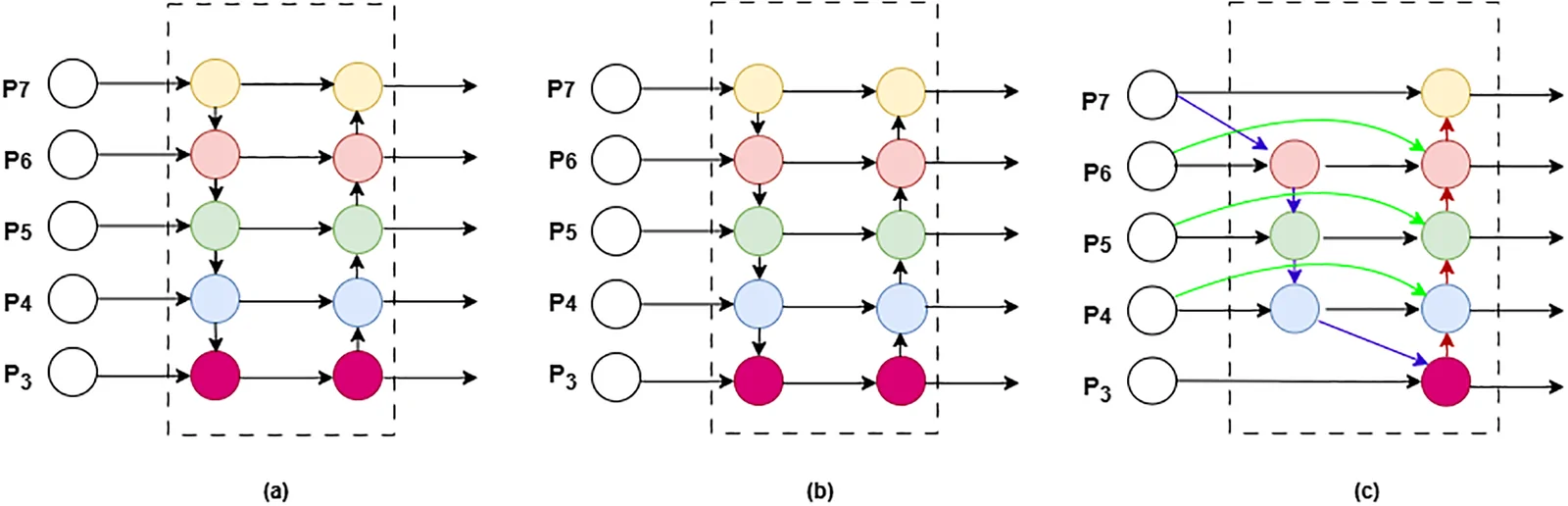
Evolution of feature fusion networks from (a) FPN and (b) PANet to (c) the BiFPN architecture, highlighting the enhanced multi-scale connectivity used to improve the detection of objects at varying scales.

As illustrated in Fig 5, unlike traditional structures such as FPN or PANet, where feature map fusion adopts a fixed pattern or unidirectional propagation, BiFPN relies on a bidirectional and adaptive approach. It enables two-way communication while automatically learning to weight the relative importance of each feature map.

In terms of application, BiFPN performs an iterative fusion of feature maps extracted at different hierarchical levels. This fusion is regulated by learned attention coefficients, ensuring that the most important features are prioritized during the aggregation process. In this way, the network manages to fine-balance the contributions of spatial details from the lower layers and global semantic information extracted from the deeper layers.

The integration of BiFPN into the neck, therefore, aims to enhance semantic consistency and the richness of multi-scale representations while maintaining reasonable computational complexity.

### Image enhancement using Contrast Limited Adaptive Histogram Equalization Technique(CLAHE)

CLAHE is a contrast enhancement technique that improves local image contrast by applying histogram equalization to small regions while limiting noise amplification. It was selected as the image enhancement method due to its effectiveness in improving visibility under challenging underwater conditions, such as low illumination, turbidity, and non-uniform lighting [29]. Unlike global enhancement methods, CLAHE operates on small regions, which allows it to enhance fine-grained features without amplifying noise excessively. Compared to Retinex-based approaches [30], CLAHE is computationally more efficient and easier to integrate into real-time detection pipelines. In addition, GAN-based enhancement methods [31], although powerful, require large annotated datasets and introduce higher computational complexity and potential artifacts. Therefore, CLAHE offers a practical trade-off between performance improvement, computational cost, and implementation simplicity, making it well-suited for the proposed YOLO-CBNet framework.

### Experimental setup

All experiments were performed on an Intel i7-12900K processor with 18 GB of RAM. The proposed model was trained and evaluated on an NVIDIA RTX 3080 GPU to ensure reproducibility of both performance and efficiency measurements.

The dataset contains 1,805 marine fish samples. It was divided into training, validation, and test sets using a standard split ratio, with 70% of the data used for training, 20% for validation, and 10% for testing. The images originate from diverse underwater scenes with varying environmental conditions. To avoid data leakage, care was taken to ensure that visually similar images or images from the same scene were not distributed across multiple splits, thereby preserving the independence of the evaluation process.

The proposed model was trained for 100 epochs using the AdamW optimizer, selected for its improved generalization capability compared to the standard Adam optimizer. To ensure a stable optimization process, a Warm-up strategy combined with Cosine Decay was employed for learning rate scheduling, allowing the learning rate to gradually increase before progressively decreasing during training.

**Fig 6:**
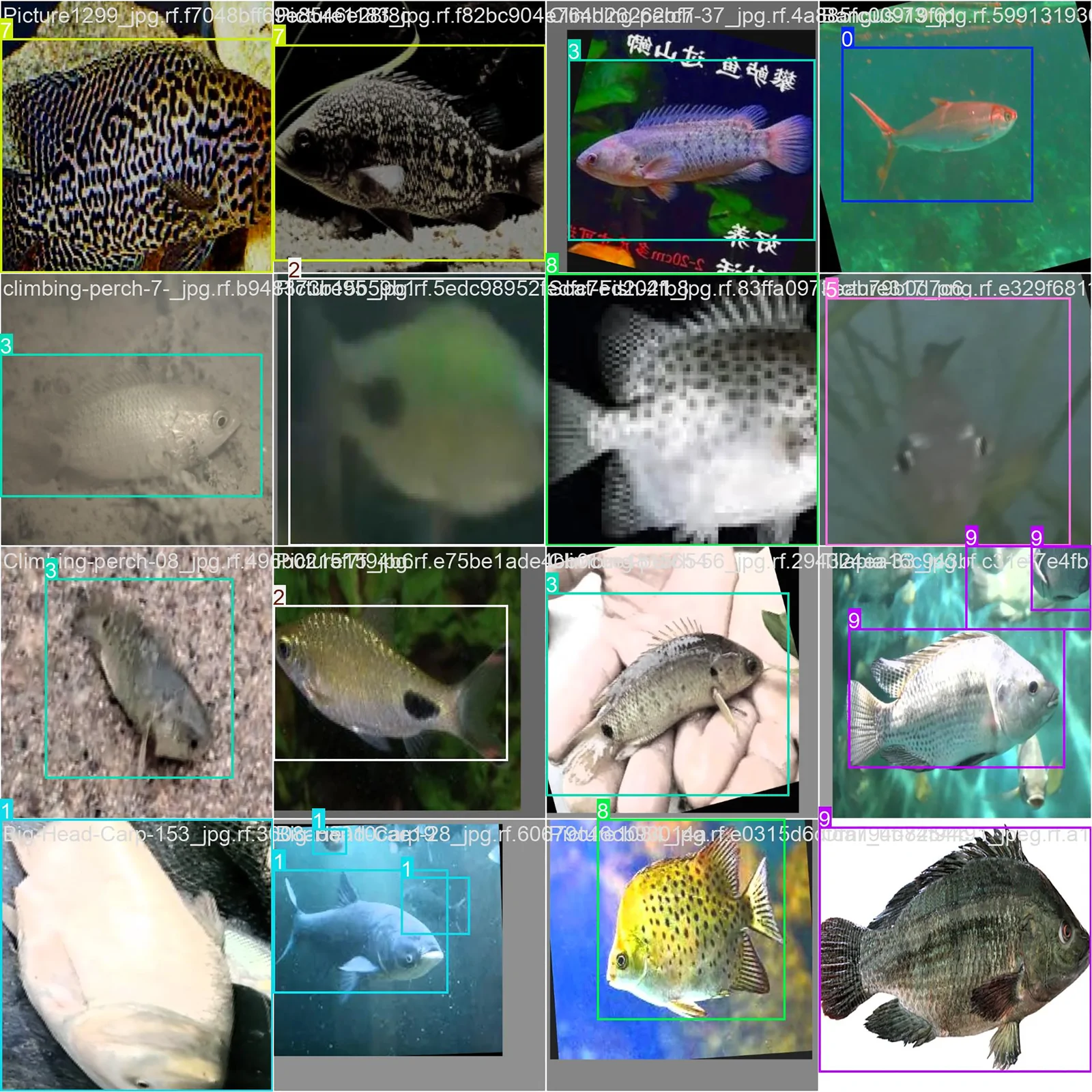
Analysis of training batch 54 content. A mosaic view of training images with ground-truth labels and boxes. The batch demonstrates strong variability in object scale, species, and image quality.

The hyperparameters were not directly taken from default YOLO settings. Instead, they were empirically tuned through a series of experiments on the validation set to achieve an optimal trade-off between accuracy and convergence stability. Table 2 provides additional details on the model configuration. The batch size was set to 128. Fig 6 shows a snapshot of batch 54 during the training process of the proposed enhanced model.

**Table 2:** YOLO-CBNet model hyperparameters.

Parameter	Value Selected
Optimizer	AdamW (lr = 3e-4, weight decay = 0.05)
LR Schedule	Warmup + Cosine Decay
Batch Size	128
Confidence Threshold	0.50
Regularization	Dropout = 0.1, StochDepth = 0.2
Epochs	100

In order to thoroughly test the robustness and generalization of our model, additional tests were performed on the five public datasets Fish-Vista dataset (Fish-Visual Trait Analysis), Tagged Fish dataset (OBSEA), Fishnet Open Images database, Fish-gres dataset, and Fish-Pak dataset. Fish-Vista dataset [32] includes about 60,000 images from nearly 1,900 fish species, designed for fine-grained visual trait analysis. It provides both species-level and pixel-level annotations for traits such as fins and barbels, enabling classification and segmentation tasks. The images, mostly sourced from museum collections, support research on morphological feature identification. The Tagged Fish dataset [33] consists of 33,805 underwater images captured between January 2013 and December 2014 at the OBSEA Cabled Observatory (4 km off the Catalan coast, 20 m depth). Fishnet Open Images database [34] contains imagery from on-board monitoring cameras in commercial fishing vessels, annotated with bounding boxes for fish species and humans across 34 object classes. The Fish-gres dataset [35] contains 8 fish species. The number of images for each species ranges from 240 to 577. The images were collected at traditional markets in Gresik, East Java, Indonesia. The resolution of the acquired images is 4160×3120 pixels before being resized to 390×520 pixels. Fish-Pak dataset [36] contains 915 high-resolution images of 6 freshwater fish species from Pakistan captured under controlled conditions using a Canon EOS 1300D camera.

## Results and discussion

This section presents and analyzes the experimental results obtained with the proposed approach. It begins with the validation of the preprocessing phase using the CLAHE technique, highlighting its impact on image enhancement. Then, a comparative analysis of the performance of the different models is provided. The section also illustrates inference visualizations produced by the trained models under various underwater conditions. Finally, the generalization capability of the proposed YOLO-CBNet model is assessed through validation on additional datasets.

### Validation of results obtained by CLAHE

**Fig 7:**
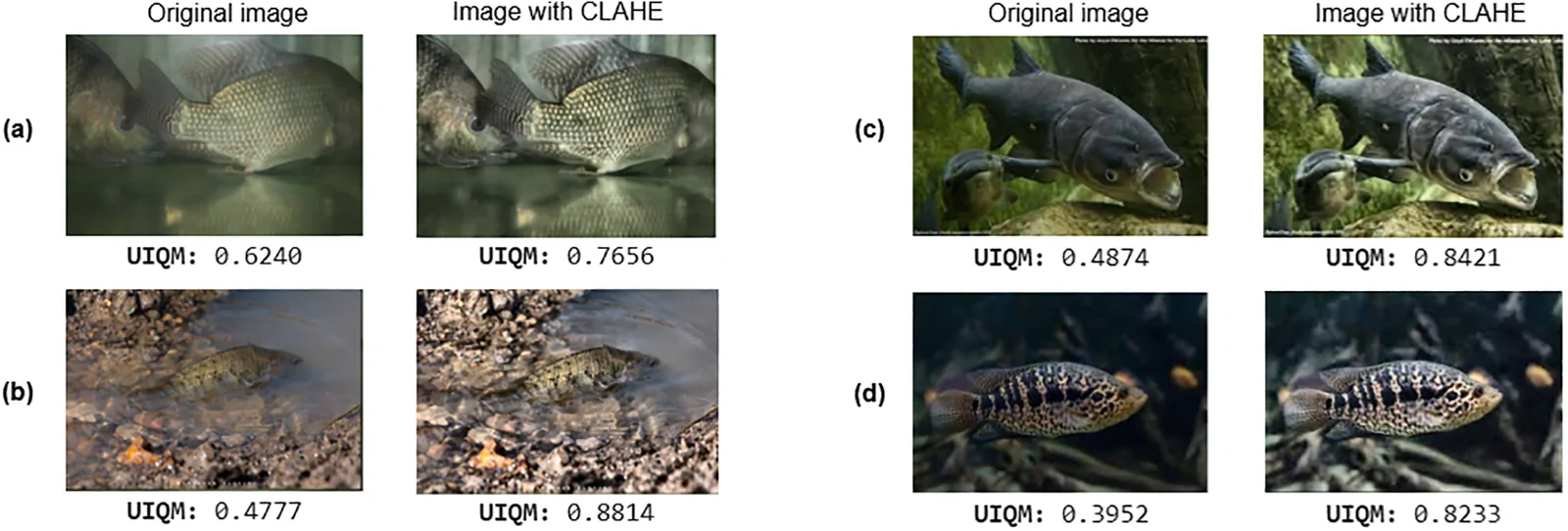
Comparison of original and CLAHE-enhanced underwater images. The results demonstrate a significant improvement in visual contrast and feature clarity across various turbid conditions (a–d). The quantitative enhancement is validated by the increased UIQM scores.

In the experimental phase, we have tested the CLAHE module separately from the YOLO-CBNet model. We used the UIQM (Underwater Image Quality Measure) metric [37], which is designed specifically for underwater images. It combines several visual aspects, such as sharpness, contrast, and natural color fidelity, allowing us to objectively quantify the improvement provided by image processing methods. Fig 7 shows four images of fish before and after applying the CLAHE technique. The UIQM value in Fig 7(a) increased from 0.6240 to 0.7656. In Fig 7(b), the UIQM value increased from 0.4777 to 0.7656. For Fig 7(c) and (d), the increases obtained are from 0.4874 to 0.8421 and from 0.3952 to 0.8233, respectively.

### Models results comparaison

**Fig 8:**
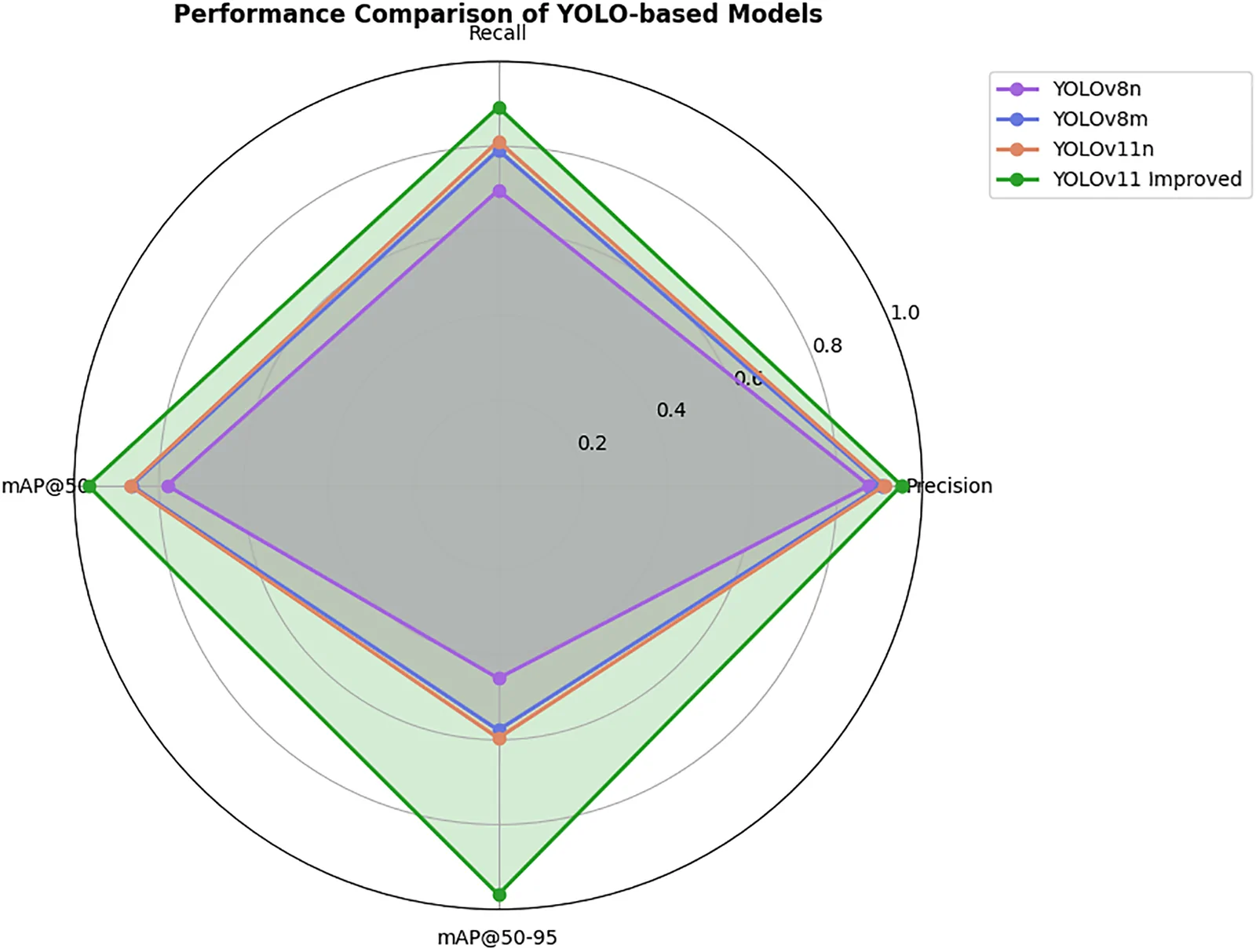
Performance comparison of YOLOv8n, YOLOv8m, YOLOv11n, and YOLO-CBNet models.

Initially, we conducted an experimental comparison between several state-of-the-art object detection models to evaluate their ability to detect and classify fish in underwater environments. The results, as presented in Table 3 and illustrated in Fig 8, show a gradual improvement in detection performance as the model architectures evolve. The YOLOv8n model achieves a precision of 87.4% and a recall of 69.6%, while YOLOv8m improves these values to 90.5% and 79.1%, respectively, with a notable gain in mAP@50 of 86.5%. The introduction of YOLOv11n further enhances the performance, reaching 91.1% precision, 81.2% recall, and 86.9% mAP@50. The recent transformer-based RT-DETR achieves competitive results, obtaining a precision of 91.9%, a recall of 84.4%, an mAP@50 of 92.2%, and an mAP@50–95 of 76.3%, demonstrating the effectiveness of transformer-based object detection for underwater fish detection. Finally, the proposed YOLO-CBNet model outperforms all the evaluated models, achieving the best results with 95.1% precision, 89.2% recall, a mAP@50 of 96.7%, and a mAP@50–95 of 96.5%.

**Table 3:** Performance comparison of state-of-the-art object detection models for underwater fish detection.

Model	Precision	Recall	mAP@50	mAP@50–95
YOLOv8n	0.874	0.696	0.781	0.455
YOLOv8m	0.905	0.791	0.865	0.577
YOLOv11n	0.911	0.812	0.869	0.596
RT-DETR	0.919	0.844	0.922	0.763
YOLO-CBNet	0.951	0.892	0.967	0.965

These improvements can be attributed to the integration of CBAM and BiFPN modules, which enhance the model’s ability to focus on informative regions and improve multi-scale feature fusion, respectively. In particular, the significant gain observed in mAP@50–95 indicates that the proposed architecture is more effective in detecting small and partially occluded fish, which are common challenges in underwater environments. Furthermore, the use of CLAHE preprocessing contributes to better contrast enhancement, improving the quality of feature extraction in low-visibility conditions.

Based on the results presented in Table 3, YOLOv11 was selected as the baseline model for our study, as it provides a better trade-off between detection accuracy and recall compared to YOLOv8 variants. This choice is further supported by its improved performance in underwater fish detection tasks.

YOLO-CBNet outperforms all other YOLO variants across all evaluation metrics, achieving the highest precision, recall, and mAP scores. To ensure a fair comparison, all models were trained and evaluated under the same experimental protocol and data split. Although the dataset size is relatively moderate (1,805 images), the observed performance improvements are consistent across all baselines, indicating stable gains rather than random variations due to data partitioning.

The representation in Fig 8 clearly confirms this improvement, illustrating the improved model’s capability and superiority in terms of the balance between accuracy, recall, and efficiency across the various evaluation metrics. These results confirm that the optimization introduced in YOLO-CBNet significantly enhances its performance in reliably and accurately detecting fish, even in complex underwater conditions.

**Fig 9:**
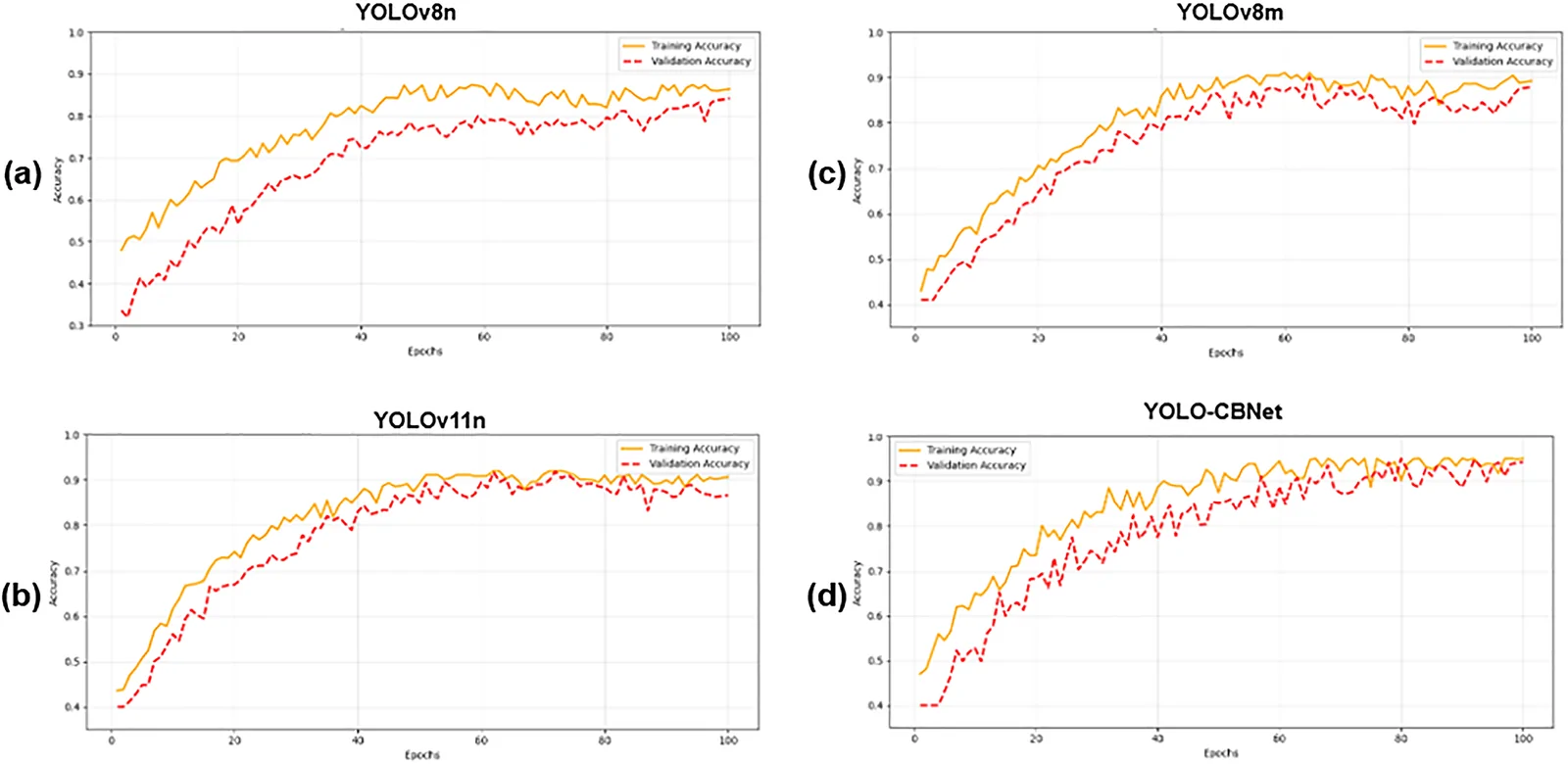
Training Progress of YOLOv8n, YOLOv8m, YOLOv11n, and YOLO-CBNet Models.

Fig 9(a) presents the evolution of the training and validation accuracy of the YOLOv8n model. The learning curves of YOLOv8m are illustrated in Fig 9(b). Fig 9(c) shows the corresponding results for YOLOv11n, while Fig 9(d) depicts the performance of the proposed YOLO-CBNet model over time. The YOLOv8n model shows steady progress, stabilizing at around 0.87 for training and 0.82 for validation, reflecting good generalization but slight under-learning. YOLOv8m achieves training accuracy close to 0.90 and validation accuracy around 0.85, showing faster convergence and a good balance between bias and variance. YOLOv11n shows increased stability and robust convergence, with both curves converging around 0.91, reflecting excellent generalization ability. Finally, the proposed YOLO-CBNet model stands out clearly with a training accuracy exceeding 0.95 and validation stabilized around 0.92, representing the best performance among the four architectures. The proximity of the training and validation curves confirms the stability of the model and the effectiveness of the joint integration of the CBAM module and BiFPN, which enhances hierarchical feature extraction and multi-level fusion.

### Ablation study

Table 4 presents a comparative evaluation of different configurations combining CLAHE, CBAM, and BiFPN with the baseline YOLOv11n model. The results show a consistent and significant improvement across all evaluation metrics as additional modules are progressively integrated. The baseline YOLOv11n model achieves a precision of 0.911, a recall of 0.812, an mAP@50 of 0.869, and an mAP@50–95 of 0.596. Incorporating CLAHE preprocessing yields a slight improvement, increasing precision to 0.913, recall to 0.817, and mAP@50 to 0.877, while maintaining the same mAP@50–95. The addition of the CBAM attention mechanism further enhances performance, reaching a precision of 0.918, a recall of 0.836, an mAP@50 of 0.901, and an mAP@50–95 of 0.661. Finally, the proposed YOLO-CBNet model, which integrates CLAHE, CBAM, and BiFPN, achieves the best performance with a precision of 0.951, a recall of 0.892, an mAP@50 of 0.967, and an mAP@50–95 of 0.965, demonstrating the effectiveness of the combined architecture.

**Table 4:** Ablation study of YOLO-CBNet components based on YOLOv11n.

Model	Precision	Recall	mAP @50	mAP @50–95	Frame rate (FPS)
YOLOv11n	0.911	0.812	0.869	0.596	76
YOLOv11n+CLAHE	0.913	0.817	0.877	0.596	75
YOLOv11n+CLAHE+CBAM	0.918	0.836	0.901	0.661	70
YOLOv11n + Retinex + CBAM+ BiFPN	0.913	0.814	0.870	0.634	64
YOLOv11n+CLAHE+CBAM +BiFPN(YOLO-CBNet)	0.951	0.892	0.967	0.965	69

Overall, the progressive integration of CLAHE, CBAM, and BiFPN leads to a substantial performance gain, with YOLO-CBNet achieving the best overall results.

Furthermore, to experimentally evaluate the impact of the image enhancement strategy, we replaced CLAHE with the Retinex enhancement method [38] while keeping the remaining architecture unchanged (CBAM and BiFPN). The Retinex-based configuration achieved a precision of 0.913, a recall of 0.814, an mAP@50 of 0.870, and an mAP@50–95 of 0.634. These results are substantially lower than those obtained with the CLAHE-based YOLO-CBNet model.

These results indicate that Retinex-based enhancement is less effective in underwater scenarios, likely due to its tendency to introduce color inconsistencies and amplify local artifacts in low-visibility conditions. In contrast, CLAHE provides more stable local contrast enhancement, which better preserves structural information essential for detection. This explains the superior performance of the CLAHE-based YOLO-CBNet configuration across all evaluation metrics. In terms of computational efficiency, the frame rate analysis shows a gradual decrease in FPS from 76 for the baseline YOLOv11n model to 75 after introducing CLAHE, and 70 when adding CBAM, indicating a moderate computational overhead due to the attention mechanism. The use of the Retinex enhancement further reduces the FPS to 64, reflecting its higher computational cost and less efficient integration with the detection pipeline. However, the proposed YOLO-CBNet still maintains a competitive inference speed of 69 FPS, demonstrating a favorable trade-off between detection accuracy and real-time performance.

### Inferences visualization with models

**Fig 10:**
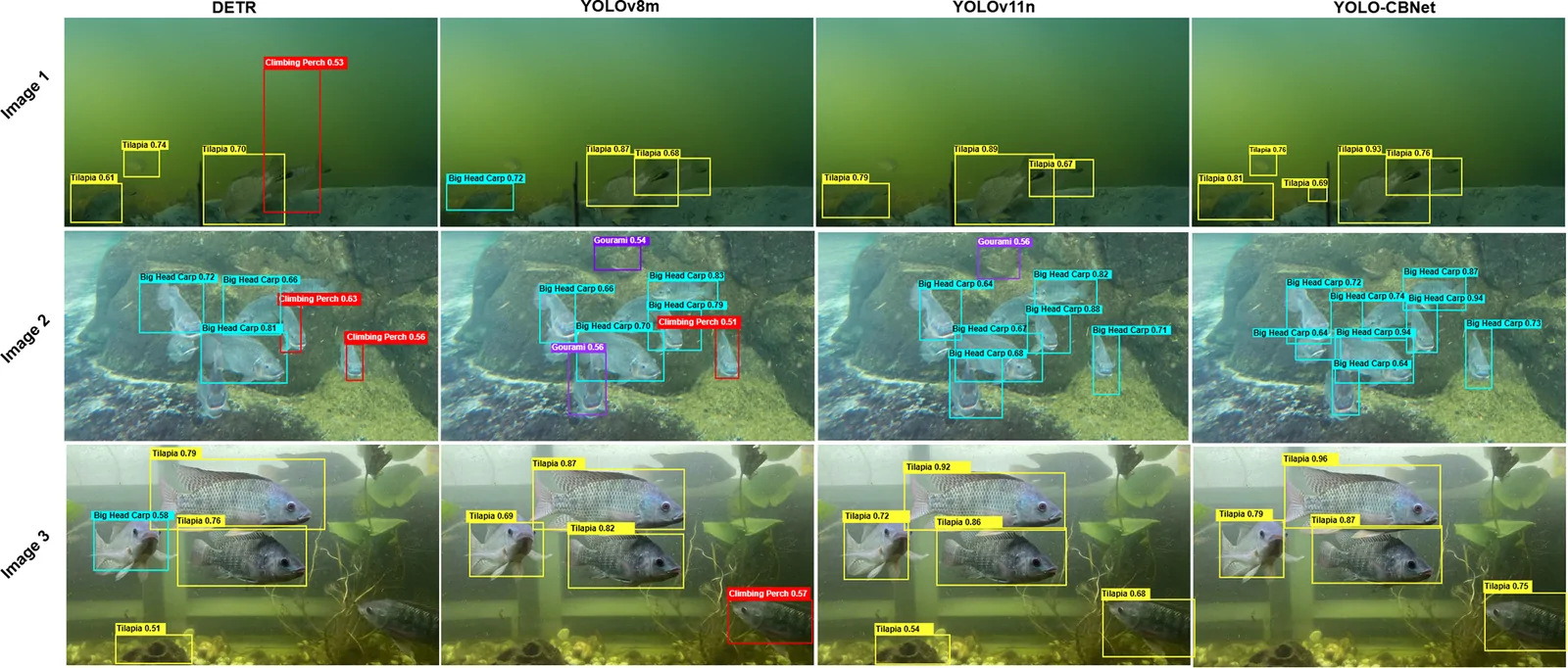
Detection and classification results of fish species using DETR, YOLOv8m, YOLOv11n, and YOLO-CBNet on a complex underwater scene.

Fig 10 shows an underwater image of various fish species. Acquisition conditions are particularly difficult due to water turbidity, low light, and the chromatic similarity between the fish’s skin and the background color. Inferences made using DETR and YOLOv8m reveal several limitations: some instances are misclassified, including a fish incorrectly identified as a Climbing Perch and Big Head Carp; furthermore, small fish or those located further away from the camera are not detected, and the bounding boxes generated remain inaccurate.

A slight improvement is observed with YOLOv11, which displays higher confidence rates and correct species classification. Nevertheless, this model continues to have difficulties in detecting small objects and in accurately adjusting bounding boxes.

**Fig 11:**
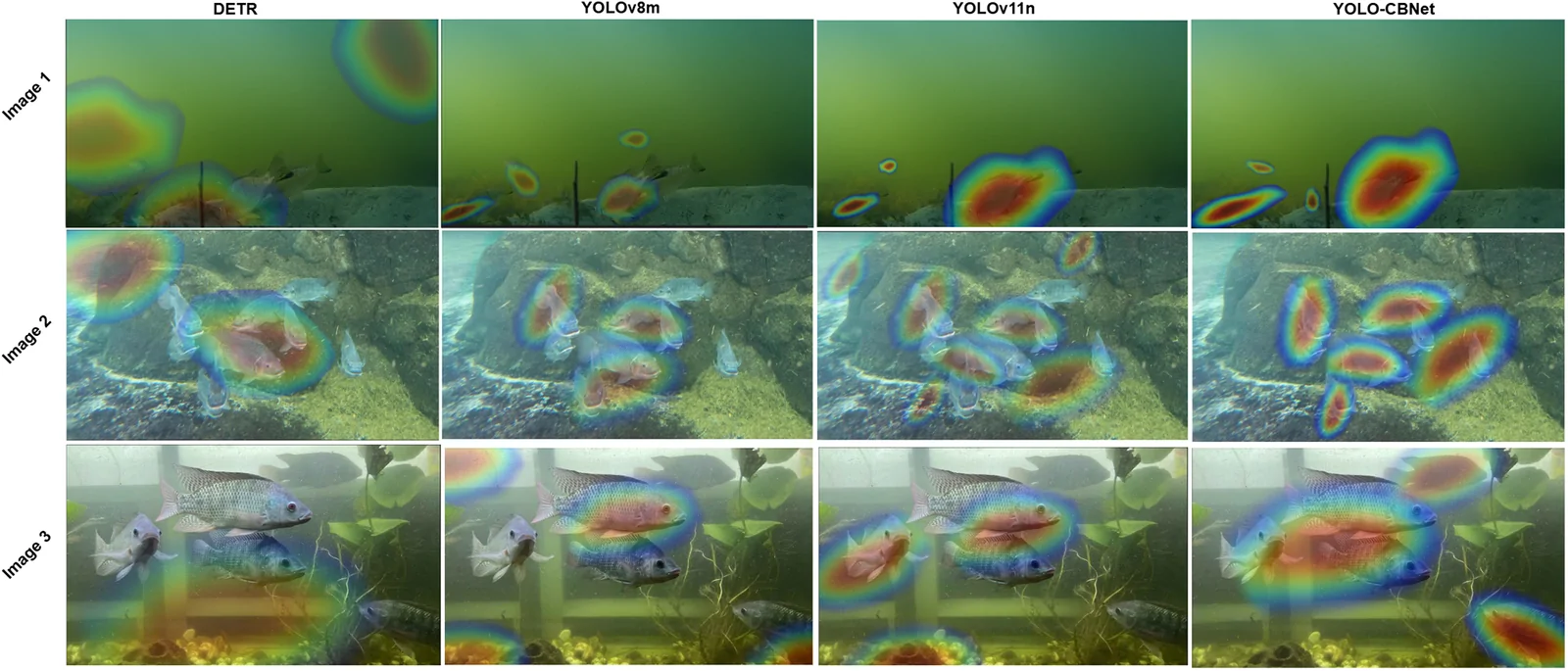
Comparison of attention heatmaps extracted with DETR, YOLOv8m, YOLOv11n, and YOLO-CBNet models.

In contrast, the proposed YOLO-CBNet model demonstrates more robust detection, including for small fish, with better-adjusted bounding boxes and better object localization. The performance of YOLO-CBNet is further confirmed in Fig 11, which shows the heatmaps generated by the different models at the time of detection. The YOLO-CBNet heatmaps are distinguished by a more precise concentration on the areas corresponding to fish, thus outperforming the other models, whose heatmaps appear more diffuse and less well aligned with the objects of interest. This indicates that the proposed model learns more discriminative spatial representations and focuses on semantically relevant regions, which contributes to its improved detection performance in challenging underwater conditions such as noise, blur, and occlusions.

### Class-wise performance analysis

**Fig 12:**
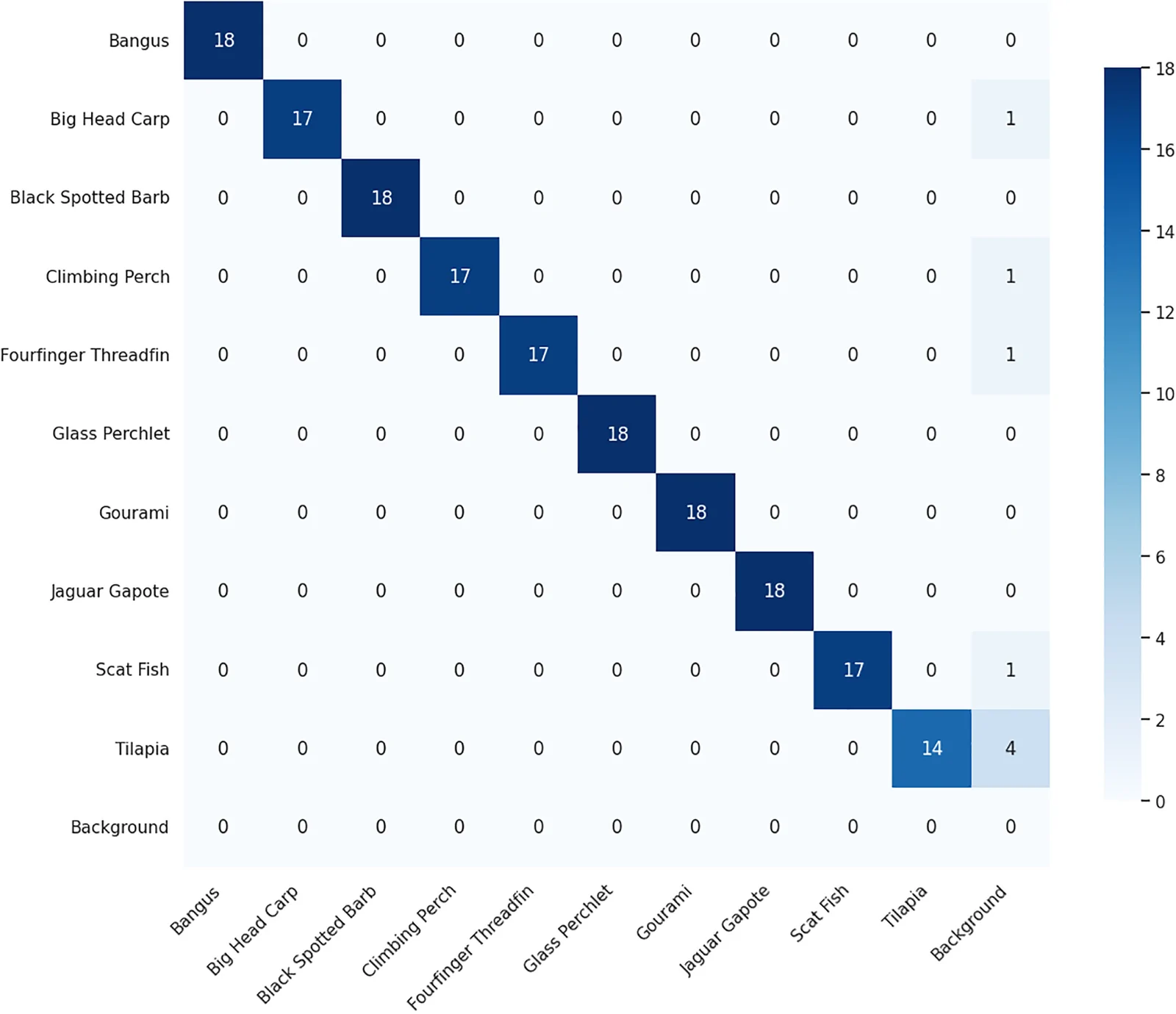
Confusion matrix of YOLO-CBNet for 10 fish species showing class-wise detection performance.

The results illustrated in Fig 12 that the proposed YOLO-CBNet model achieves strong class-wise discrimination, with most fish species being correctly classified, as indicated by the dominant diagonal values. Specifically, classes such as Bangus, Black Spotted Barb, Glass Perchlet, Gourami, and Jaguar Gapote achieve perfect classification without errors, highlighting the robustness of the model for these species. The overall confusion matrix shows a strong diagonal dominance, indicating that most predictions are correctly assigned to their respective classes, with only a very small number of misclassifications.

However, the confusion matrix reveals that the main source of errors is not inter-class confusion between fish species, but rather misclassification towards the background class. A small number of instances from Big Head Carp, Climbing Perch, Finger Threadfin, and Scat Fish are incorrectly classified as background, indicating occasional missed detections. This issue is more pronounced for Tilapia, where 4 samples are misclassified as background, suggesting that this class is more challenging due to its visual characteristics or environmental conditions.

Overall, the absence of significant confusion between fish species confirms the strong discriminative capability of the model, while the limited errors observed are mainly related to detection difficulty rather than class ambiguity. These results are consistent with the high precision and mAP values reported in the quantitative evaluation.

A closer inspection of the misclassified samples reveals that most failure cases occur under challenging underwater conditions, including low illumination, water turbidity, partial fish occlusion, and strong overlap between individuals. In these situations, the model occasionally fails to detect the fish and predicts the background class instead. These errors are primarily associated with degraded visual quality rather than confusion between different fish species. Despite these challenging scenarios, the number of failure cases remains limited, confirming the robustness of the proposed YOLO-CBNet model in realistic underwater environments.

### Inference speed and computational complexity analysis

To evaluate the efficiency of the proposed YOLO-CBNet model, we measured its inference performance, computational complexity, and memory consumption on a standard workstation equipped with an NVIDIA RTX 3080 GPU. Specifically, we report the frame rate (FPS), latency (ms), number of parameters (Param), number of floating-point operations (GFLOPs), and peak GPU memory usage, providing a comprehensive assessment of the model in terms of detection performance, computational cost, and resource requirements. In addition to the YOLO-based detectors evaluated in earlier experiments, we included DETR and a ViT-only detector as reference transformer-only models to assess inference speed and latency. DETR provides a relevant benchmark for fully transformer-based detection approaches, allowing a broader comparison of speed-accuracy trade-offs. The ViT-only detector employs a Vision Transformer as the sole backbone for feature extraction and object prediction, without relying on any YOLO-based components, serving as a baseline to evaluate the contribution of integrating YOLO and the CBAM module in the proposed YOLO-CBNet architecture. Table 5 presents the detection accuracy, computational complexity, memory consumption, and inference performance of YOLO-CBNet in comparison with the baseline models, including YOLOv8, ViT-only detectors, and DETR-based transformer detectors. YOLO-CBNet achieves the highest detection accuracy among the evaluated models. Its inference speed, measured at 69 FPS with a latency of 18.2 ms per image, lies between the high-speed YOLOv11n model and the slower transformer-only approaches, demonstrating a measurable balance between speed and accuracy in numerical terms. In addition to inference performance, the proposed YOLO-CBNet model was trained for 6241 seconds on an NVIDIA RTX 3080 GPU for 100 epochs. This training duration remains reasonable given the added architectural components. The observed increase in computational cost for YOLO-CBNet compared to YOLOv11n is mainly due to the integration of CBAM and BiFPN modules, which introduce additional feature refinement and multi-scale fusion operations. However, the model remains significantly more efficient than transformer-based architectures such as DETR, which suffer from higher computational complexity and lower inference speed. This confirms that YOLO-CBNet maintains a favorable balance between accuracy improvement and computational efficiency, making it suitable for real-time underwater fish detection applications. Overall, YOLO-CBNet offers a good balance between accuracy and computational efficiency. It improves detection performance while only slightly reducing the frame rate from 76 FPS to 69 FPS. This reflects a classic trade-off between accuracy and speed, with a limited impact on real-time performance.

**Table 5:** Inference speed and detection accuracy of different models.

Model	mAP @0.5	Frame rate (FPS)	Latency (ms)	Param (M)	GFLOPs	Peak GPU Memory (GB)
YOLOv11n	0.8690	76	12.4	12.55	6.8	2.6
DETR	0.7681	16	52.3	22.83	12.24	6.8
ViT-only detector	0.8114	24	47.8	11.63	6.34	2.9
YOLOV-CBNet	0.9670	69	18.2	13.89	7.21	3.1

### Statistical validation and reproducibility analysis

To address concerns about the statistical reliability and reproducibility of the YOLO-CBNet model, we conducted five separate training runs using different random seeds, while maintaining the same dataset split and experimental settings. This allows us to assess the model’s stability under stochastic training conditions and provides a more reliable estimate of performance variability. Table 6 displays the results in terms of mAP@50 for the five test sets, for both the YOLOv11n model and the proposed YOLO-CBNet model.

**Table 6:** Performance across five independent runs (mAP@50).

Run	YOLOv11n	YOLO-CBNet
Run 1	0.8690	0.9670
Run 2	0.8710	0.9680
Run 3	0.8680	0.9650
Run 4	0.8720	0.9670
Run 5	0.8670	0.9660

According to the five separate runs shown in Table 6, YOLOv11n achieved an average mAP@50 of 0.8694, while the proposed YOLO-CBNet model achieved an average mAP@50 of 0.9666, demonstrating both superior accuracy and great stability across different random initializations.

In addition, 95% confidence intervals (CI) were used to quantify the reliability of the estimated performance. The intervals obtained ranged from [0.8668; 0.8720] for YOLOv11n to [0.9652; 0.9680] for YOLO-CBNet, indicating low variability and high consistency across the five independent runs.

To evaluate the statistical significance of the proposed approach in greater detail, a paired t-test was conducted on the five independent runs, revealing that the performance improvement of YOLO-CBNet over YOLOv11n is statistically significant (*p* < 0.01) and that it is unlikely to be due to random variation.

The results confirm that the performance improvement of YOLO-CBNet is not due to random variation but is consistent across several separate training cycles. The low standard deviation further demonstrates the stability and robustness of the proposed approach when faced with different stochastic initializations. Overall, this analysis addresses concerns regarding statistical significance, confidence intervals, and reproducibility, thereby reinforcing the reliability of the presented experimental results.

### Hyperparameter sensitivity analysis

To assess the stability of the proposed YOLO-CBNet model with regard to key learning and inference hyperparameters, a sensitivity analysis was performed. This study examines the impact of changes in the learning rate schedule, batch size, and confidence threshold on detection performance. For each hyperparameter, the other parameters were kept the same, and only the parameter under study was modified. As presented in Table 7, the model was evaluated using the mAP@0.5 to measure the consistency of performance across different configurations. The results demonstrate that YOLO-CBNet maintains stable performance regardless of the hyperparameter configuration. This indicates that the proposed model is not very sensitive to moderate variations in training and inference parameters, which demonstrates its robustness and reliability.

**Table 7:** Hyperparameter sensitivity analysis of YOLO-CBNet.

Hyperparameter	Setting	mAP@0.5 (%)
Learning rate schedule	Warmup + Cosine Decay Cosine Decay Step Decay	0.9670 0.9581 0.9643
Batch size	128 64 32	0.9670 0.9622 0.9594
Confidence threshold	0.50 0.25 0.75	0.9670 0.9501 0.9623

### Robustness evaluation under challenging conditions

To assess the robustness of the proposed YOLO-CBNet model, additional experiments were conducted on degraded versions of the test dataset. Two common image degradations were considered: Gaussian noise [39], and Gaussian blur [40], which simulate challenging underwater imaging conditions such as sensor noise, motion blur, and poor visibility.

The trained YOLO-CBNet model was directly evaluated on the degraded datasets without any retraining or fine-tuning. Table 8 presents the results in terms of mAP@0.5, used to quantify the impact of each type of degradation on model accuracy.

**Table 8:** Robustness evaluation of YOLO-CBNet under different image degradation conditions.

Condition	mAP@0.5 (%)
Original images	0.9670
Gaussian noise	0.9198
Gaussian blur	0.9302

The results show that YOLO-CBNet maintains strong robustness under different image degradation conditions. Although a slight decrease in performance is observed when Gaussian noise and Gaussian blur are introduced, the overall drop in mAP@0.5 remains limited.

### Model validation on other datasets

To evaluate the generalization performance of the proposed YOLO-CBNet model, we conducted an external evaluation on four public fish datasets, in addition to the main dataset used for training and internal evaluation. The YOLO-CBNet model was trained exclusively on the main dataset. The four additional datasets were used solely for testing purposes, without any retraining or fine-tuning. These external datasets contain images captured under varied and complex underwater conditions, including low resolution, poor water visibility, lighting variations, and visual similarities between fish species and the background. These characteristics make them relevant benchmarks for evaluating the model’s performance in realistic scenarios. The objective of this evaluation is to measure the model’s transferability and generalization ability when applied to completely unknown data. As shown in Table 9, the YOLO-CBNet model achieves high and stable performance across all external datasets, thereby confirming its robustness when faced with different data distributions.

**Table 9:** Performance of YOLO-CBNet on different fish datasets.

Dataset	Fish Species	Precision (%)
Fish‑Vista dataset [32]	Oreochromis niloticus, Scomber japonicus, Lutjanus argentimaculatus, Seriola dumerili, Thunnus albacares, Mugil cephalus, Chanos chanos, Epinephelus coioides, etc.	96.41
Tagged Fish dataset [33]	Diplodus vulgaris, Oblada melanura, Chromis chromis, Diplodus sargus, Coris julis, Sarpa salpa, Serranus scriba, Symphodus tinca, etc.	97.18
Fishnet Open Images database [34]	Albacore tuna, Yellowfin tuna, Skipjack tuna, Bigeye tuna	92.65
Fish-gres dataset [35]	Chanos chanos, Johnius trachycephalus, Nibea albiflora, Rastrelliger faughni, Upeneus moluccensis, Eleutheronema tetradactylum, Oreochromis mossambicus, Oreochromis niloticus	98.04
Fish-Pak dataset [36]	Ctenopharyngodon idella, Cyprinus carpio, Cirrhinus mrigala, Labeo rohita, Hypophthalmichthys molitrix and Catla catla	91.16

The results obtained with our proposed YOLO-CBNet architecture show a significant improvement over previous studies, and it is useful to analyze this improvement from three perspectives: detection accuracy, model complexity, and generalization to complex conditions in aquaculture environments. Table 10 presents a comparison between our approach and several other studies on the same task. YOLO-CBNet clearly stands out from previous methods with remarkable accuracy of 95.1%, surpassing models such as FishDet-YOLO (89.5%), SCMYOLO (90.6%) and YOLO-Fish (76%). This improvement can be explained by the effectiveness of the joint integration of attention modules (CBAM) and BiFPN multi-scale fusion, reinforced by CLAHE preprocessing adapted to the specific characteristics of the underwater environment.

**Table 10:** Comparative performance and complexity of fish detection models.

Work	Approach	Dataset	Precision(%)	Model Complexity (GFLOPs)
[41]	Fish detection in real marine environments, YOLO-Fish1/2 lightweight architectures.	4,505 images (DeepFish), 1,800 images (OzFish)	76.56	~6.5
[42]	MobileNetV3 backbone + YOLOv5-based lightweight underwater fish detector.	Internal dataset	90.60	2.3
[43]	Enhanced YOLOv8 with UEM and Mamba-C2f modules for long-range dependencies.	DUO & RUOD datasets	88.80 - 89.50 (mAP)	19.5
**Our work**	Integration of CLAHE preprocessing and YOLOv11 backbone improved with CBAM + BiFPN.	1,805 images (10 species) + evaluation on five public datasets.	95.10	7.2

In terms of complexity, our model strikes an excellent balance between performance and computational cost. YOLO-CBNet offers an architecture based on an improved version of YOLOv11, suggesting controlled complexity. By comparison, SCMYOLO displays remarkable computational efficiency of 2.3 GFLOPs but at the cost of slightly lower accuracy with 90.6%, while FishDet-YOLO achieves 19.5 GFLOPs for 89.5% accuracy. Our approach has a value of 7.2 GFLOPs, representing a particularly favorable compromise between accuracy and efficiency.

### Limitations and failure cases

Furthermore, the robustness observed in difficult underwater conditions in the presence of turbidity, low light, or overlapping fish attests to the relevance of our architectural choices. Nevertheless, certain points deserve to be qualified: the dataset used consists of 1,805 images, a relatively small amount compared to major benchmarks such as DeepFish, which contains nearly 40,000 images. This limitation may influence generalization to more varied contexts. Some other limitations remain in highly challenging underwater conditions. In particular, the model can be misled in cases of extreme turbidity, where visibility is severely degraded and object boundaries become unclear. In addition, certain fish species can change their skin color to blend with the background, making them difficult to distinguish from the surrounding environment, especially when located in the background. Furthermore, the detection of very small fish remains challenging due to their limited visual features and reduced spatial resolution. These factors may lead to missed detections or inaccurate predictions in complex scenes.

### Practical implications

Despite these limitations, the proposed model demonstrates strong potential for real-world deployment. The proposed model, which jointly performs fish detection and classification, shows strong potential for deployment in real aquaculture systems. In practical environments, underwater cameras can be used to continuously monitor fish populations within cages. The detection capability enables precise localization of fish, while the classification module provides species-level identification, which is essential in multi-species farming scenarios. This combined functionality allows intelligent feeding strategies to be implemented, where feed distribution can be adapted according to species behavior and density, thereby reducing feed waste and operational costs. Moreover, the model can support real-time monitoring and decision-making by providing continuous insights into fish activity, population structure, and environmental adaptation. Its high accuracy and real-time performance make it particularly suitable for integration into smart aquaculture systems.

## Conclusion

In this study, we proposed a novel hybrid architecture, YOLO-CBNet, for accurate underwater fish detection. The model combines CBAM to emphasize the most informative spatial and channel features with BiFPN for efficient multi-scale feature fusion. Additionally, the preprocessing step using CLAHE was applied to enhance image contrast, improving the detection of fish in challenging conditions such as turbidity, low illumination, and background similarity.

Experimental results demonstrated that YOLO-CBNet outperforms baseline YOLOv11n model, achieving improvements of +4.0% in precision, and +9.8% in mAP@50. These results confirm the effectiveness of the proposed architecture in handling complex underwater environments.

These findings highlight the potential of attention-based architectures combined with contrast enhancement for intelligent aquaculture monitoring. However, we recognize limitations related to the size and diversity of the dataset, which may not fully capture the variability of real underwater scenarios. Future work will focus on expanding the dataset to include more diverse environmental conditions and fish species, optimizing the model for lightweight inference on embedded devices, and exploring multi-task extensions, combining detection with fish health assessment and behavior monitoring to advance sustainable aquaculture practices.
